# Sensor-based assessment of social isolation in community-dwelling older adults: a scoping review

**DOI:** 10.1186/s12938-023-01080-4

**Published:** 2023-02-27

**Authors:** Shehroz S. Khan, Tiancheng Gu, Lauren Spinelli, Rosalie H. Wang

**Affiliations:** 1 KITE, University Health Network, 550, University Avenue, Toronto, M5G 2A2 Canada; 2 Department of Occupational Science and Occupational Therapy, University of Toronto, 160-500 University Avenue, Toronto, M5G 1V7 Canada

**Keywords:** Ambient sensors, Assessment, Older adults, Sensors, Social isolation, Wearable sensors

## Abstract

Social isolation (SI) is a state of low social interaction with peers associated with various adverse health consequences in older adults living in the community. SI is most often assessed through retrospective self-reports, which can be prone to recall or self-report biases and influenced by stigma. Ambient and wearable sensors have been explored to objectively assess SI based on interactions of a person within the environment and physiological data. However, because this field is in its infancy, there is a lack of clarity regarding the application of sensors and their data in assessing SI and the methods to develop these assessments. To understand the current state of research in sensor-based assessment of SI in older adults living in the community and to make recommendations for the field moving forward, we conducted a scoping review. The aims of the scoping review were to (i) map the types of sensors (and their associated data) that have been used for objective SI assessment, and (ii) identify the methodological approaches used to develop the SI assessment. Using an established scoping review methodology, we identified eight relevant articles. Data from motion sensors and actigraph were commonly applied and compared and correlated with self-report measures in developing objective SI assessments. Variability exists in defining SI, feature extraction and the use of sensors and self-report assessments. Inconsistent definitions and use of various self-report scales for measuring SI create barriers to studying the concept and extracting features to build predictive models. Recommendations include establishing a consistent definition of SI for sensor-based assessment research and development and consider capturing its complexity through innovative domain-specific features.

**Supplementary Information:**

The online version contains supplementary material available at 10.1186/s12938-023-01080-4.

## Introduction

With the advent of modern technologies and changes in how people interact with one another, social isolation (SI) has become a pervasive societal issue. One definition of SI in the literature is an observable lack of social contact with others [1–3]. SI is related to but distinct from loneliness, which is a subjective feeling and a cognitive evaluation of the quantity and quality of relationships [4].

SI is particularly troubling for older adults and research has been increasingly focused on SI in those aged 55 and older [5, 6]. Experiences relevant to the aging life course such as living alone, bereavement, relocation, retirement, and chronic health conditions have been correlated with patterns of SI [7, 8]. Various reports [8, 9] indicate that worldwide more than 25% of adults over the age of 65 are socially isolated. Furthermore, the ongoing COVID-19 pandemic has significantly exacerbated concerns with SI in older adults [10]. SI in older adults can be associated with depression, dementia, poor cardiovascular health, overall well-being and mental health, and premature death [11, 12]. SI is also considered a risk factor for elder abuse and may increase fear of crime and theft; therefore, it may make older adults less likely to participate in social activities [13]. Older immigrants, minority ethnic groups and low-income older adults are at risk of becoming lonely and likely to have fewer social interactions [14]. Prolonged SI has harmful economic, health, and social consequences for society [12].

Despite the negative health and social consequences of SI, it is not adequately managed in primary care settings [15, 16]. SI is not routinely assessed in primary care settings and there is a lack of established best practices for assessment in the community [15, 17]. One reason may be that primary care clinicians feel that their main responsibilities are to address biomedical issues rather than social issues. Additionally, considering their heavy workloads and the limited time they have in direct contact with individual patients, they may not have a way to reliably observe and identify social isolation [17]. Assessments for SI are often conducted through self-report. There exist several validated clinical scales that may be used, such as the Lubben Social Network Scale (LSNS) [18], Duke Social Support Index [19], and the Social Disconnectedness Scale [20]. These scales are questionnaires that quantify how frequently individuals are in contact with their social connections, which often include information on frequency of phone calls, number of friends or relatives that the individual is regularly in contact with, whether the individual lives alone, their marital status, and how often they engage in religious or club-based activities [3, 18]. A limitation of these scales is that they involve subjective assessment of an objective phenomenon, which introduces recall and self-report biases that may affect assessment accuracy. These scales ask for retrospective information, and not intended to detect or predict SI onset. Lack of motivation to self-report also creates barriers to assessing SI. Personalities that are more or less likely to disclose SI or those who are more likely to participate in research may skew reports, as can stigma associated with SI [21]. Men, in particular, are less likely to report SI because of the stigma around being isolated and lonely [22]. The biases inherent in current SI assessments may reduce the identification of isolated individuals, thereby limiting the provision of services to mitigate SI related problems.

Considering the consequences of SI for older adults, lack of routine community-based assessment, and limitations of current approaches, it is beneficial to develop objective SI assessments based on ambient and wearable sensors that can be completed regularly and sustainably in the community. Collecting these data over time for older adults who are at risk for SI allows for proactive and prospective detection of SI, which is important for timely and comprehensive health assessment and delivery of evidence-based individualized interventions. The data collected from different sensors may capture a person’s indoor and outdoor behaviours, interactions with the environment (e.g., opening or closing refrigerator), and physiological and mobility indicators. Novel features can then be extracted from this rich multimodal dataset and fed to machine learning models that can detect and predict the onset of or changes in SI. The need for an automated longitudinal approach is not just because patients do not self-report or that they are unreliable or biased, but also that clinicians are failing to help as well because they do not have the time, resources and awareness to do it and that a longitudinal approach would be beneficial to identify the onset of SI [15, 16].

Prior research has suggested that objective data collected through sensors can help to make inferences regarding SI using predictive modelling [23]. Previous studies show that time spent napping, length of stay in the living room, amount of physical exercise, and time spent out of the home correlate with self-reported SI [1, 24, 25]. Wearable devices, such as smartwatches and smartphones, embedded with accelerometers and Global Positioning Systems (GPS), are often used to measure an individual’s physical, mobility, and activity levels [26]. Sensor technology has great potential to collect objective data that may be relevant to the identification and elucidation of SI, such as social contacts with others and time spent doing activities in the community.

One recent non-systematic literature review [23] and another scoping review [27] aimed to survey and synthesize research on technology applications for the detection and monitoring of SI. Bouaziz et al. [23] presented an in-depth survey of both wearable and non-wearable (ambient) sensors, software and algorithms that have been applied in the monitoring of older adults’ daily activities, with many conducted in laboratory settings. They specifically focused on on meal-taking and mobility from which the risk of SI may be inferred at a later stage that was not detailed in the review. The scoping review by Qirtas et al. [27] examined passive sensing including smartphones, wearable devices and ambient sensors to detect loneliness and SI. This review covered a general population that comprised younger adults (34% or 10 of 29 included studies), older adults (41% or 12 of 29 included studies) and mixed age (17% or 5 of 29 included studies) groups. While both reviews have defined SI and loneliness as distinct from one another, neither review has attempted to distinguish them in their selection of articles for review. Indeed Qirtas et al. studied SI and loneliness as a unified construct. This distinction is essential to preserve for theoretical and practical reasons, considering information on SI may be more observable, and therefore more accessible using sensor-based detection approaches compared to subjective phenomenon such as loneliness. Both reviews, encompassing a range of ages, concluded that the study of sensor-based assessment of SI and loneliness is in its infancy.

To our knowledge, there has not been a review examining the use of sensors in SI assessment for older adults living in the community and that connects existing approaches for the assessment of SI, namely self-report scales or direct observation data, with objective sensor-based measures to validate this new assessment approach. There is also a lack of clarity regarding the application of various sensors and their data in assessing SI and methods used to develop these assessments, information that will be important to ultimately create meaningful and useful SI assessments for this population. To understand the current state of research and to make recommendations for the field moving forward, we conducted a scoping review. The aims of the scoping review were to map the types of sensors (and their associated data) that have been used for objective SI assessment, and identify the methodological approaches used to develop the SI assessment. Using review results, we synthesize current understandings and knowledge gaps to be addressed in future research to make this form of assessment a reality.

## Results

Eight articles were included in the review. The articles ranged in disciplines from public health, gerontology, information technology, and sleep science. Table 1 summarizes information from the articles. The age range of participants in our review varied from 50 years to 91 years. There is no clear pattern of definition of SI, self-reported measure or type of sensing modalities across these age groups.

**Table 1:** Summary of articles included in scoping review to examine sensor-based assessment of social isolation in community-dwelling older adults

Study (author and year)	Study location	Population sample	Definition of SI from study	Self-reported measure of SI	Type of sensor technology	Study’s conclusion
Martinez et al. [28], 2017	Mexico	7 participants between 60–74 years old, 2 M and 5 F	The lack of contact and interaction with others	LSNS	Motion sensor: Sensor beacons Smartphone: mobile applications	The predictive model demonstrated 100% accuracy for determining the level of SI in a 7 person sample
Schrempft et al. [29], 2019	UK	267 participants aged 50+, 136 M and 131 F	The absence of regular contact with family and friends and lack of involvement in social organizations	Unique scale created for study	Actigraphy: wrist-mounted accelerometer (triaxial actigraph)	A higher level of SI correlates with lower level of physical activity and a higher level of sedentary behaviours
Tully et al. [30], 2019	Denmark, Spain, Germany, UK	1360 participants 65+ years old across 4 countries; 517 M and 843 F	The quantitative measure of social relationships and contacts	LSNS-6	Actigraphy: hip-mounted accelerometer (ActiGraph GT3X+)	Physical activity was not associated with SI. Sedentary behaviours may be linked to SI in older adults. Sedentary behaviours and physical activity were found to be not significant predictors of loneliness
De Koning et al. [31], 2019	UK	112 participants 65+ years old living across 23 rural villages or isolated dwellings in Wiltshire, South West England. 54 M and 58 F	Infrequent social contact	Social Capital Module (SCM) – 3 items based on SI	Actigraphy: waist-mounted Actigraph (GT3X)	There were no associations between loneliness, SI and objectively measured physical activity
Herbolsheimer et al. [32], 2017	Germany	1162 participants 65+ years old from 1 city who were German speaking, not institutionalized, and not using a wheelchair, 665 M and 497 F	Restricted social networks; a perceived lack of social support by family, friends, and neighbours	LSNS-6	Actigraphy: leg-mounted accelerometer (ActivPAL single-axis accelerometer)	Low indoor physical activity was associated with being socially isolated from family and low outdoor physical activity was associated with being socially isolated from friends and neighbours
Goonawardene et al. [33], 2017	Singapore	46 participants between 60 and 91 years old, mostly of Chinese descent (87%), in Singapore. 19 M and 27 F	A lack of interpersonal contacts with society	LSNS	Motion sensor: passive infrared (PIR) motion sensors	The average time spent outside home is associated with the social loneliness level, social network score and the overall SI level of the elderly and the time spent in the living room is positively associated with the emotional loneliness level
McCrory et al. [34], 2014	Ireland	4888 participants 50+ years old, 2242 males and 2646 females	Lack of social connectedness or social support	Berkman Social Network Index	Physiological sensor: electrocardiography	There is a negative association between social network size and resting heart rate
Benson et al. [35], 2020	USA	759 participants aged 58–85. 356 M and 403 F	Lack of contact with others, measured through self-report and objective measures of social connections	SI (expanded version of social disconnectedness score by Cornwell and Waite for the NSHAP study; 9 variables)	Actigraphy: wrist-mounted actigraph (Actiwatch Spectrum),	SI and loneliness had a low correlation to each other. Both were associated with actigraph measures of more disrupted sleep (wake after sleep onset, % sleep) but not associated with actigraph total sleep time. Increased loneliness associated with more insomnia symptoms and shorter sleep duration (assessed by single question); SI was not. More isolated individuals spend longer time in bed

SI: Social Isolation; LSNS: Lubben Social Network Scale; LSNS-6: 6-item Lubben Social Network Scale; NSHAP: National Social Life, Health and Aging Project; M: male; F: female

### Sensor technologies, data measured and features extracted

Table 2 details the sensors, measured data and features extracted in the assessment of SI. Features used for assessing SI were grouped into four categories—sleep metrics, physical activity, phone communication, and physiology. Among the eight articles, five used actigraphy and two used motion sensors. One study used a mobile application to record the frequency and duration of phone communication behaviours. One of the articles examined the relationship between SI and resting heart rate using electrocardiography.

**Table 2:** Summary of sensor technologies, measured data and features used in assessment of social isolation

Types of sensors	Features	References
Motion sensor and actigraphy	Sleep metrics: total sleep time, the sum of all epochs scores as wake during the sleep interval, percent sleep	Goonawardene 2017 [33] Benson 2021 [35]
	Physical activity: duration of stay time in each area at home, room-level movement, number of places where the older adult stayed, types of movement (walking, quiet standing, sitting/lying) , Time out of home	De Koning et al. [31], Martinez et al. [28], Schrempft et al. [29], Herbolsheimer et al. [32], Goonawardene et al. [33], Tully et al. [30]
Smartphone	Phone communication (via phone monitor application): number of incoming calls, number of outgoing calls, the average duration of family calls, number of calls from friends, the average duration of outgoing calls to family, average outgoing messages to friends	Martinez et al. [28]
Physiological Sensor	Resting heart rate	McCrory et al. [34]

*Association between features and self-reported SI measures* Five of the eight articles used physical activity as the objective feature to indicate SI, though findings regarding the relationship with SI are inconsistent. Schrempft et al. [29], Herbolsheimer et al. [32], and Goonawardene et al. [33] concluded that there is a negative association between SI and physical activity. In contrast, Tully et al. [30] and De Koning et al. [31] concluded that there is no significant association between SI and physical activity. Both Tully et al. [30] and De Koning et al. [31] suggested that their inconsistent findings might be due to either small sample size or the fact that the participants in the sample had low risk of SI. Three studies investigated the relationship of SI with other sensor features. McCrory et al. [34] concluded a negative relationship between resting heart rate and social network size—a concept based on social contact, which aligned with the review’s definition of SI. The study by Benson et al. [35] found a negative association between SI and total sleep time. The study by Martinez et al. [28] evaluated the accuracy of a predictive model of SI, but there was no discussion about the associations between SI and other variables.

*Types of statistical modelling* Among the eight articles, seven studied the association between self-reported SI data and one uses predictive modelling. These seven studies used descriptive modelling to determine whether SI is associated with specific behavioural or physiological measures [36]. The study by Martinez et al. [28] focused on detecting an older adult’s self-reported SI level based on sensor data [37]. They used phone communication variables and physical activity variables as predictors of an individual’s LSNS score and reported 100% accuracy. Predictive models are valuable in identifying an individual who is experiencing SI; however, it may be difficult to understand the complex (non-linear) interplay of features to detect SI.

**Table 3:** Self-reported measures of social isolation

Self-report measures of SI	Description	Scoring	Articles
LSNS and LSNS-6 [18]	Questionnaire created specifically for use with older adult populations. Assesses SI based on perceived connectedness to friends, family and neighbours	10-item LSNS or 6-item LSNS-6. Items are scored from 0 to 5. Summed scores range from 0 to 30, with scores less than 12 indicating high risk for SI. Measures size, closeness, and frequency of contacts in their social network. The LSNS- 6 was developed from the LSNS for brevity	Herbolsheimer et al. [32], Goonawardene et al. [33], Tully et al. [30], Martinez et al. [28]
Three questions on SI from the Social Capital Module [31]	Assess social and civic participation across 3 categories of SI from relatives, friends and neighbours	Items are scored from 1 to 4, with 1 indicating engagement most days and 4 indicating engagement less than once per month. SI was indicated for each category if the total score was less than or equal to 2	De Koning et al. [31]
Unique scale (created for authors’ study) [29]	Assess monthly contact with others, participation in social activities	Items are scored 1 point if answered as less than monthly contact with family and friends and if they did not participate in social activities. Total scores range from 0-4, with a score of 3 or 4 suggesting SI. Scored according to contact with children, other family members, and friends. 1 point received if they do not participate in organizations such as social clubs, religious groups, and committees	Schrempft et al. [29]
Social Disconnectedness Score [20]	Measures: Social network size, Social network range, Proportion of social network members in same household. Average frequency of interaction with network member, Number of friends, Frequency of attending meetings of an organized group, Socializing with friends and relatives, Frequency of volunteering, attending religious service. Participants also indicate how many friends they have	The average of the 8 variables is calculated and then reversed to indicate disconnectedness (SI). Total scores range from -1.30 to 2.34, with higher score indicates greater-than-average social disconnectedness and greater perceived isolation	Benson et al. [35]
Berkman Social Disengagement Index [38]	Measures: Presence of spouse, Monthly visual contact with 3+ relatives and close friends, Yearly non-visual contact with 10+ relatives and close friends, frequent attendance at religious services, membership in other groups, regular participation in recreational social activities	Total score between 0 and 4. A higher score indicates greater social connection	McCrory et al. [34]

SI: Social Isolation; LSNS: Lubben Social Network Scale; LSNS-6: 6-item Lubben Social Network Scale

### Methodological approach to SI assessment development

Table 3 summarizes the five self-report measures of SI included in the articles. The review demonstrates that different articles use different nuanced definitions of SI. Two broad categorizations of social isolation scales emerged from this analysis. First, the scales that focus on social contact with family and friends, and second, the scales that goes beyond social contact and take into consideration other factors, including older adults’ participation in social, religious or other outdoor activities. The LSNS-6 scale [18] measures perceived social support of an older adult received by family and friends, it assesses size, closeness and frequency of contact of a person’s social network. De Koning et al. [31] scale focuses on how often older adults meet their friends and relatives and speak to their neighbours face-to-face. From sensing perspective, accelerometers, indoor motion sensors and other physiological sensors (e.g., sleep mat) can collect important data to capture one or more of these factors. The other three scales go beyond the social contact, to include the presence of a specific family member in the household, size of social networks, and frequency of participating in specific social activities. Schrempft et al. [29] scale assigns points if the person had less than monthly contact with their family and friends, and if they participate in social activities. The Social Disconnected Scale [20] assesses the lack of connectedness to other individuals and social groups. However, it is more comprehensive in comparison to Schrempft et al. [29]. It considers the number of people in a person’s social network and frequency of contact with them, number of people living in the same household and connection with other individuals outside of household. It also accounts for the number of friends, and social activities outside the home. Berkman Social Disengagement Index [38] considers the presence of a spouse, monthly visual contact with three or more relatives or friends, yearly non-visual contact with 10 or more relatives or friends, and attendance at religious or social activities. In this scale, the significance of visual and non-visual contact with three and ten relatives/friends is not clear. The later three scales highlight the importance of activities outside of the household. From sensing perspective, the traditional accelerometer and inside home sensors may not be adequate and using (wearable) devices with in-built GPS can be a better to understand their out-of-the-home social behaviour [39]. The GPS data can also further shed light if they are mobile within home only or going outside and visiting different places.

The five scales found in the review used different numbers and types of questionnaire. The LSNS-6 [18] and De Koning et al. [31] scales contain six and three questions, whereas the number of questions in Social Disconnected Scale [20] and Berkman Social Disengagement Index [38] are eight and six. Schrempft et al. [29] did not clearly stated the number of questions asked in their scale. Due to varying number of questions and social dimensions captured by these scales, they add up to different values with varying interpretation, and it is difficult to directly compare them. In the Social Disconnected Score [20] (used by Benson et al. [35]) and the scale used by Schrempft et al. [29], a higher value indicated higher risk for SI, whereas in the other three scales the opposite holds true.

Previous studies show that older adults have fewer social interactions and a small social network in comparison to younger adults [40, 41]. However, it is not clear if the social network size of older adults drastically changes over a short period of few weeks or months, unless there are adverse personal or health circumstances. Outdoor social activities, on the other hand, can be good indicator of their well-being but it may vary due to various conditions, including weather, festivals or other social events (whether alone or in company with family or friends). Therefore, we suggest using social isolation scales containing both social contacts/networks and social activities and they should be administered for a longer duration to arrive at a better assessment.

## Discussion

In a sample of 2506 articles, only eight articles were determined to meet the inclusion criteria for this review. This indicates that the topic of assessment of SI within a community-dwelling older adult population using sensors, while potentially beneficial, is a topic in its infancy. The reason for the small number of included studies was also likely due to our restrictive inclusion/exclusion criteria, which limited to studies that focused on SI as an objective determinant and included older adults living in the community and excluding those living in congregate living environments such as nursing homes. This unique focus makes results from this review unlike other published reviews and can be helpful to re-orient future directions for those involved in community-based older adult care. Due to these major differences in research question, search strategy and analysis, there are only two studies (i.e. Goonawardene et al. [33] and Martinez et al. [28]) that overlaps between ours and Qirtas et al. [27]’s reviews. Below, we discuss the complexities in SI, its assessment, our results, highlighting the gaps in the current state of knowledge, summarize recommendations for future research, and present limitations of our review.

### Defining SI

While we selected a definition of SI for the purposes of setting up inclusion criteria for this scoping review, establishing a precise definition was a repeatedly encountered barrier. In the reviewed literature, there is inconsistency and overlap within the definitions of SI and loneliness. SI is sometimes explained as “the objective characteristics of a situation” and to what degree someone is alone [4]. In other research, it is taken as a state in which one needs social contact but does not have the means to acquire it [42]. Meanwhile, some definitions of loneliness claim it as something that “can be measured objectively in terms of the number of friends and social contacts” [43], while others define it as a subjective feeling that occurs when social relationships are deficient in some way [4] quantitatively or qualitatively [44]. Mansfield et al. [45] have provided a review of these definitions and concluded that while these concepts of SI and loneliness are distinct concepts, there are many definitions in use that make them often interchangeable, and their phenomena have complex interactions in the human experience. This makes the process of assessing these constructs difficult because what is being assessed or measured may change depending on the definition, the clinical scales used, and the approach to assessment. Additionally, the definition of SI often overlaps with terms such as ‘social disconnectedness’ [20], which can lead to divisions in language used in research.

Thus, when encountering articles in the screening process of the scoping review, a critical eye and discussion (among team members) was given to each article that discussed concepts related to SI or loneliness. We noticed that some articles mentioned SI in the title or abstract but did not meet our inclusion criteria of an objective measure related directly to SI. For example, the study by Eldib et al. [46] tested the potential of a video camera to monitor the number of visitors, which can be an objective measure of SI. However, the study did not relate the objective measure to a self-report measure of SI. Additionally, many articles were screened to full-text review due to their use of the concept of ‘objective loneliness’ often being similar to SI. However, they were later excluded due to the construct measurements being based on perception (such as “How often do you feel that you lack companionship?”) rather than objective and observable data. For example, the study by Robins et al. [1] investigated the association between physical activity and SI in community-dwelling older adults; however, they only used a self-report measure of SI and lacked an objective sensor component. One excluded SI article made use of the UCLA Loneliness scale [47] while measuring behaviours using sensors [48]. Comparatively, the questions within the SI questionnaires included in our review are objective and do not rely on the perceptions of subjective experience.

### Sensors used in SI assessment

Results from the review suggest that the evidence to use various sensors (and features extracted from them) for SI assessment are inconsistent. Other research looking at social and physical activities has shown that these relationships are complex [31]. This inconclusiveness in the literature may be a result of unclear definitions and relationships between the constructs of physical activity and SI. In addition, the use of electrocardiography for detecting SI might be limited, because of the wide range of factors that can influence heart rate and introduce confounding variables. Also, an individual’s heart rate can vary depending on sex, age, lifestyle, and other health factors. The variations in resting heart rate can pose challenges to using it as an objective measure of social isolation. Another challenge is from the feature extraction perspective. The SI assessments may be done at the beginning, end or some mid-points during the study. However, the sensor data are collected on a continuous basis. Therefore, it is not clear what should be the window for feature extraction, e.g., a day, a week, 2 weeks, or so.

This review shows a lack of diversity in ambient and wearable sensors being used to assess SI in older adults. Interesting forms of technology are emerging in the fields of observing behaviours and social connections, such as RFID and microphones [49], smart home sensors that can observe temperature, interactions with the environment and interactions with others [48, 50, 51] and E-textiles [52]. These types of sensors have potential for collecting relevant information for the assessment of SI and are worth investigation.

Ultimately sensor-based assessment of SI may benefit from multi-sensor systems that can capture different types of data, including mobility, sleep, indoor and outdoor motion, social behaviours [39]. It will further enable increase accuracy of assessment and predictive algorithms.

### Methodological approach to SI assessment development

The scoping review found more descriptive studies than predictive studies.

In recent times, machine learning and deep learning approaches have improved the state-of-the-art in many applications, including human activity recognition [53, 54]. However, this review revealed only one such study for assessing SI by Martinez et al. [28]. This clearly shows a lack of predictive algorithm integration to further the study of objective assessment of SI. The small amount of data collected by these studies could be a constraining factor in developing generalizable predictive models. SI is not an instant event; instead, it develops over time and long-term studies spanning several months are required. However, executing such studies can bring their own challenges, in terms of privacy, ethical approvals, data collection protocols and transfer mechanism, data storage and security, sensor malfunction, battery requirements, rejection of technology, participant attrition and financial constraints [55, 56]. A major future direction is the development of scalable digital health solutions that can stream, store, collate and analyse large multimodal data over a longer time period in a cloud-framework [39].

### Strengths and limitations

We acknowledge that the definitions used within this scoping review may have limited the scope of included articles; therefore, some may have been missed or excluded based on our definition of SI, or language choice and lack of explicit focus on SI in the screened articles. The use of different questionnaires to assess SI may have also made it difficult to capture all relevant articles for this reason. Non-English language material was excluded due to cost and time to translate material, which the authors acknowledge may have limited the inclusion of relevant articles.

### Comparison with previous work

The work by Qirtas et al. [27] used general population and a unified construct to study SI and loneliness, which is different from our focus. Their conclusions were around validation of loneliness/SI scales, developing machine learning algorithms, costs of devices, sensors’ battery life, privacy and ethics. These are great directions; however, they are general pointers towards any digital health assessment and intervention platform. The review by Bouaziz et al. [23] is more focused on identifying activities of daily living as indicator of SI. They further provided general recommendations around using different sensors, algorithms and data that can improve such activities to improve detection of SI. We summarize our recommendations and conclusions below.

### Recommendations

Based on the knowledge gaps identified in this review, several recommendations can be made to guide future research into the development of sensor-based assessment of SI. Addressing these gaps is essential for future researchers to systematically establish knowledge of SI assessment among older adults living in the community and explore a broad range of potential technologies for assessing SI. (1) Establish a consensus definition of SI that focuses on observable behaviours that are more directly linked to SI, affirming that SI as a phenomenon separate from loneliness, social disconnectedness, and perceived social isolation, and agreeing on a set of behaviours relevant to SI. An agreed-upon definition and set of behaviours can then be used to in future studies to develop self-report and sensor-based assessments for SI. (2) Patient-reported measures continue to be important elements of overall assessment and care planning in health settings. To further enhance validity and decrease the risk for recall or self-report bias in the development of sensor-based assessments, it may be beneficial to include event recording where people track their own behaviours in real time or where researchers make direct observations as part of the development process. (3) Explore other types of sensors, such as door sensors, smart watches, cameras (RGB, depth, infrared), sleep mats, GPS and voice activated smart devices (e.g., Google Home, Amazon Alexa) as additional ways of collecting information related to SI. (4) Develop an in-depth theoretical and empirical framework that maps the diversity of features that can be extracted from sensor data that may be used to evaluate SI more directly such as activities that people do that involve others, e.g., phone use, time spent around other people indoors or outdoors. An important consideration is the amount of sensor data that is deemed necessary to extract informative features. (5) The perception of older adults for technology may be different from other populations. Therefore, for future developments, involvement of older adults in the co-design of relevant systems will improve their usability and adoption for better outcomes.

## Conclusions

This review suggests that despite being an important problem among older adults living in the community, SI and its assessment using technology has not received the desired attention from the research community. Major gaps were identified in terms of the definition of self-reported and objective measures of SI. Future research can benefit from diversifying behavioural and social features extracted from sensors to draw upon predictive models. Among the eight articles included, only two articles mentioned sensors that are not motion sensors or actigraphy. In the selected articles, few feature types were explored, such as phone communication, heart rate, blood pressure, and body temperature. More investigation is required to extract other meaningful features from the reviewed and other sensing modalities. Further exploration through long-duration studies is needed to determine whether features are significantly associated with SI, which will eventually improve the overall accuracy of predictive models.

## Methods

We selected the scoping review methodology (rather than other forms of knowledge synthesis) because it is best suited to address exploratory research questions and enable the mapping of key concepts, types of evidence, and gaps in research related to a defined area or field [57]. Our review followed the five steps outlined by Arksey and O’Malley [58] and advanced by Levac et al. [59]. The steps we completed were the following: (1) identifying the research question, (2) identifying relevant literature, (3) study selection, (4) data extraction, and (5) gathering and reporting findings. The sixth optional step, consultation, was not completed due to our review’s primarily descriptive purpose.

### Identifying the research question

We sought to answer the following questions: *What sensor technologies are being used in the objective assessment of SI in community-dwelling older adults? and What methodological approaches have been applied to develop SI assessments?*

### Inclusion/exclusion criteria

*Participants’ age* Articles were included if the participants were aged 55 and older, as this cohort is considered ‘older’ regarding computing and related technology [60]. We only included articles involving wider age groups if at least 65% of their included populations were older adults and their results were distinguished from young or middle-aged adult participants.

*Health conditions* We excluded articles with a focus on comorbidities such as falls risk, cardiovascular disease, schizophrenia, or dementia to reduce extraneous variables and ensure the results of the scoping review related to a general group of older adults.

*Living situations* We included articles where data collection was conducted with individuals who lived in independent housing such as a house, townhome, or apartment. We excluded articles where data were collected from individuals who lived in retirement homes, group homes, or long-term care facilities, where additional care support was provided. While there is a potential that older adults living in congregate facilities may be at higher risk for SI owing to poorer overall health and functional abilities, most older adults are living in the community where there is also a potential for underreporting of SI among these individuals [61].

*Social isolation* Consistent with our focus on SI (as opposed to loneliness or other related construct), we aimed to include articles only if they focused on SI,. which for the purposes of this review was defined as an observable lack of social contact with others [1–3]. Given inconsistencies in use of terms and definitions in the current literature, we applied a functional and pragmatic approach such that if a term other than SI was used in an article, but the definition applied was consistent with the definition used in this review, the article was included. We included articles using self-report scales that incorporated elements of SI in their description and assessment. That is, if some of the questions asked in the scale were relevant to SI, the article was considered for inclusion. We excluded articles if all of the questions in the scales focused on measurements of subjective experiences, such as ‘feeling isolated from others’ and ‘having others around them that they trust’ [47, 62], rather than measures of SI such as “how many of your friends do you see or talk on the phone each week.” [38].

*Sensors* Articles were included if they used any type of ambient or wearable sensor that can collect data to indicate SI, such as information about an individual’s activities, location, interactions with their environment, and/or physiology were included. Examples include ambient sensors and ‘smart home’ devices with contact sensors or light sensors. Wearable and physiological devices can include body worn garments, belts, chest straps, and smart watches or phones that can collect GPS and radio frequency identification; heart rate monitors, accelerometers or actigraphy, respiration monitors, EEG, video cameras and microphones.

Additionally, articles were included if they were published in any year, involved humans, and published in the English language. Primary sources were included from any discipline if they were peer-reviewed journal articles or conference papers.

### Identifying relevant articles

To collect a comprehensive list of primary studies, two authors conducted an initial search of the literature using electronic databases (Medline, CINAHL, IEEE and Google Scholar). Additionally, reference lists of relevant articles, key journals and conferences were reviewed for topics related to SI and technology. Index terms of relevant articles were noted. An academic health sciences librarian from the University of Toronto was consulted to establish and execute an effective search strategy from keywords and index terms. A search strategy document from the University of Alberta that detailed geriatrics-related terms was also consulted to refine the search [63]. A finalized search strategy was translated across Medline, Scopus, CINAHL, PsychINFO, and IEEE. As there are syntax differences among the databases, the search strategy had to be translated for each database, which resulted in search terms that varied depending on the database. (Refer to Additional file 1: Appendix for search terms and strategy.) The search was executed on August 28, 2020 and re-executed on April 10, 2021, and 1 article was added to the full-text review after screening.

### Study selection

**Fig. 1:**
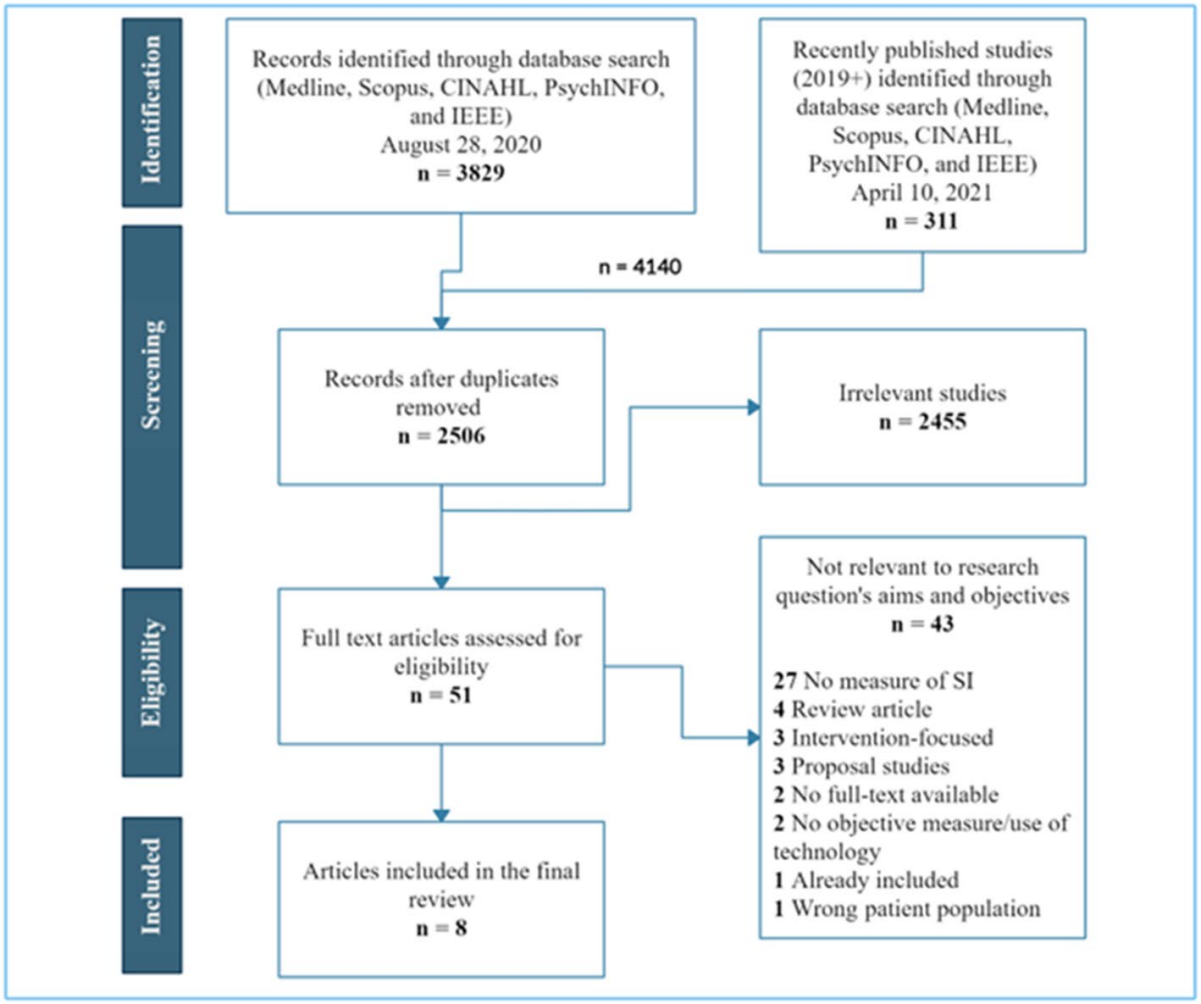
PRISMA flow diagram illustrating the search strategy, including article identification, screening, and selection which resulted in 8 articles included in the scoping review

An initial search identified several articles discussing the use of sensors in SI and loneliness research. To keep the focus of the scoping review to relevant journal articles, inclusion and exclusion criteria were established based on the initial search results, and the criteria were refined throughout the study selection process. Covidence, an online software, was used to manage study selection [64]. Two reviewers (TG and LS) applied the inclusion and exclusion criteria to the articles identified using the search strategy. Titles and abstracts were screened for relevance in Covidence. Full-text review was conducted by both reviewers if the article was deemed potentially relevant or unclear. Reviewers were required to reach agreement for all articles reviewed, and a third and fourth reviewer (SK and RW) were consulted to resolve any discrepancies. A PRISMA flow diagram was generated using the results from screening in Covidence (see Fig. 1 for PRISMA chart). Of the original 2506 articles captured by the search strategy, 51 articles proceeded to review in full by two reviewers (TG and LS), of which eight met the criteria for inclusion and proceeded to data extraction.

### Charting the data

Data from each relevant article were extracted and coded into an Excel spreadsheet by two reviewers (TG and LS) and cross-examined for consistency. Information deemed to be relevant to address our research question comprised the article’s year of publication, and the study’s geographical location, research question(s), number of participants, participant characteristics (including population context), definition of SI, self-reported measures of SI used, types of sensors, features extracted from sensor data and conclusion of the study. Additional information of interest that was extracted were the type of statistical modelling (descriptive or predictive), machine learning algorithm employed (if applicable), and statistical analysis tool. Once data were extracted, they were summarized for further analysis (see Tables 1, 2 and 3).

### Gathering and reporting the findings

The authors reviewed and analysed the data tables to identify trends within the included articles to synthesize what is known and the knowledge gaps to be addressed in future research and outline possible recommendations for the field. Documentation of this review adheres to the Preferred Reporting Items for Systematic reviews and Meta-Analyses extension for Scoping Reviews (PRISMA-ScR) reporting standards, except for the protocol not being registered [65].

## Supplementary Information

**Additional file 1: Appendix. **Search terms and strategy applied to databases for the conducted scoping review.

## Data Availability

The search strategy and keywords used in this scoping review are available in the Additional file 1.
