# Supplementary material for: Sensor-based assessment of social isolation in community-dwelling older adults: a scoping review
Source: Biomed Eng Online. 2023 Feb 27;22:18. doi: 10.1186/s12938-023-01080-4 (PMC9969951; doi:10.1186/s12938-023-01080-4)
Supplement: Supplementary file 1 — Additional file 1: Appendix. Search terms and strategy applied to databases for the conducted scoping review. [file 12938_2023_1080_MOESM1_ESM.docx]

# APPENDIX

Search terms and strategy applied to databases for the conducted scoping review.

| Database and Search terms |
| --- |
| MEDLINE  1. Technology/ or Monitoring, Ambulatory/ or Telemetry/ or Wearable electronic devices/ or Fitness Trackers/ or Monitoring, physiologic/ or Myography/ or Smart Glasses/ or Smartphone/ or Mobile Applications/ or Radio Frequency Identification Device/ or in-home monitoring/  2. ((motion adj3 sensor*) or (motion adj3 detect*) or (contact adj3 sensor*) or (contact adj3 detect*) or (ambient adj3 sensor*) or (ambient adj3 detect*) or (ambient adj3 assess*) or (audio adj3 microphone) or (telemonitor* or "remote monitor*")).ti,ab,kw,kf.  3. (gps or "global positioning system" or "enviro* sensor*" or "video camera*" or "computer vision*" or "call log*" or "phone log*" or "activity log*" or acceleromet*).ti,ab,kw,kf.  4. (smartphone or "smart watch" or "smart home" or "internet us*").ti,ab,kw,kf.  5. Internet of Things/  6. ("internet of things" or "IoT").ti,ab,kw,kf.  7. Respiration/ or Respiratory Rate/ or Heart Rate/ or Body Temperature/  8. (respirat* or "heart rate*").ti,ab,kw,kf.  9. electroencephalography/ or brain waves/ or alpha rhythm/ or beta rhythm/ or gamma rhythm/ or theta rhythm/  10. ((sleep adj3 pattern) or (sleep adj3 cycle) or (sleep adj3 track)).ti,ab,kw,kf.  11. ((ambient adj3 temperature) or (environment* adj3 temperature) or (body adj3 temperature)).ti,ab,kw,kf.  12. 1 or 2 or 3 or 4 or 5 or 6 or 7 or 8 or 9 or 10 or 11  13. exp Aged/ or exp Middle Aged/ or exp Geriatrics/ or exp Aging/  14. ("old* adult*" or elder* or (senior* not "high school") or aged or aging or ageing).ti,ab,kw,kf.  15. 13 or 14  16. Social Isolation/ or Loneliness/  17. ("social isola*" or "social engag*" or "social connect*" or "social disconnect*" or "social support*" or lonel*).ti,ab,kw,kf.  18. 16 or 17  19. 12 and 15 and 18  20. limit 19 to (english language and humans) |
| CINAHL  1. (MH “Geriatrics”) OR (MH “”Aging”) OR (MH “Aged”) OR (MH “Aged, 80 and Over”)  2. TI(“old* adult*” OR elder* OR senior* OR age*) AND AB (“old* adult*” OR elder* OR senior* OR age*)  3. S1 or S2  4. (MH “Loneliness”) OR (MH “Social Isolation”)  5. TI (“social isolat*” OR “social engag*” OR “social disconnect*” OR “social support*” OR “lonel*”) AND AB (“social isolat*” OR “social engag*” OR “social disconnect*” OR “social support*” OR “lonel*”)  6. S4 OR S5  7. MH (“ambulatory monitoring”) OR (MH “Monitoring, Physiologic”) OR (MH “Actigraphy”) OR (MH “Telemetry”) OR (MH “Polysomnography”) OR (MH “Electrocardiography”) OR (MH “Blood Pressure Monitoring, Ambulatory”) OR (MH “Blood Glucose Monitoring”) OR (MH “Myography”)  8. (MH “Wearable Sensors”) OR (MH “Fitness Trackers”) OR (MH “Smart Glasses”) OR (MH “Smartphone”) OR (MH “Mobile Applications”) OR (MH “Radio Frequency Identification”)  9. (MH “Respiration”) OR (MH “Respiratory Rate”) OR (MH “Heart Rate”) OR (MH “Body Temperature”) OR (MH “Electroencephalography”) OR (MH “Brain Waves”)  10. (TI((motion OR contact OR ambient) N2 (sensor* OR detect*)) OR AB((motion OR contact OR ambient) N2 (sensor* OR detect*)) )OR (TI (audio N2 microphone) OR AB(audio N2 microphone) )  11. TI(gps OR “global positioning system” OR “enviro* sensor*”) OR “video camera*” OR “computer vision*” OR “call log*” OR “phone log*” OR “activity log*” OR accelerometer) OR AB(gps OR “global positioning system” OR “enviro* sensor*”) OR “video camera*” OR “computer vision*” OR “call log*” OR “phone log*” OR “activity log*” OR accelerometer)  12. TI(smartphone OR “smart watch” OR “smart home” OR “internet us*”) OR AB(smartphone OR “smart watch” OR “smart home” OR “internet us*”)  13. TI(sleep N2 (pattern OR cycle OR track)) OR AB(sleep N2 (pattern OR cycle OR track))  14. TI((ambient OR environment* OR body) N2 temperature) OR AB((ambient OR environment* OR body) N2 temperature)  15. S7 OR S8 OR S9 OR S10 OR S11 OR S12 OR S13 OR S14  16. S3 AND S6 AND S15 |
| Scopus  1. Sensors:  ( "social isola*" OR "social disconnect*" OR "social engag*" OR lonel* ) AND ( "older adult*" OR aged OR elder* OR aging) AND ( sensor OR "motion sensor*" OR "contact sensor*" OR "magnetic sensor*" OR "light sensor*" )  2. Gadgets  ("social isola*" OR "social disconnect*" OR "social engag*" OR lonel*) AND ("older adult*" OR aged OR elder* OR aging) AND ("smart home*" OR smartphone* OR "smart watch*" OR "smart glasses" OR "gps" OR "global positioning system" )  3. Tracker and EEG  ( "social isola*" OR "social disconnect*" OR "social engag*" OR lonel*) AND ( "older adult*" OR aged OR elder* OR aging) AND ( wearable* OR "fitness tracker*" OR "electroencephalography" OR " brain wave*" OR "sleep pattern" )  4. Physiology and rfid  ( "social isola*" OR "social disconnect*" OR "social engag*" OR lonel*) AND ( "older adult*" OR aged OR elder* OR aging) AND ( "body temperature*" OR "ambient temperature*" OR vibration OR humidity OR rfid )  5. Other unused keywords:  ( "social isola*" OR "social disconnect*" OR "social engag*" OR lonel*) AND ( "older adult*" OR aged OR elder* OR aging) AND ( "computer vision*" OR "internet us*" OR "activity log*" OR "call log*" OR "heart rate" OR "sleep cycle" )  6. ( "social isola*" OR "social disconnect*" OR "social engag*" OR lonel*) AND ("older adult*" OR aged OR elder* OR aging) AND ("ambient sensor*" OR "video camera*" OR "sleep track" OR acceleromet* OR "radio frequency identification device") |
| PsychINFO  1. monitoring/ or telemetry/ or wearable devices/ or smartphones/ or mobile phones/ or technology/  2. ((motion adj3 sensor*) or (motion adj3 detect*) or (contact adj3 sensor*) or (contact adj3 detect*) or (ambient adj3 sensor*) or (ambient adj3 detect*) or (ambient adj3 assess*) or (audio adj3 microphone) or (telemonitor* or "remote monitor*")).ti,ab,id.  3. (gps or "global positioning system" or "enviro* sensor*" or "video camera*" or "computer vision*" or "call log*" or "phone log*" or "activity log*" or acceleromet*).ti,ab,id.  4. ((ambient adj3 temperature) or (environment* adj3 temperature) or (body adj3 temperature)).ti,ab,id.  5. (smartphone or "smart watch" or "smart home" or "internet us*").ti,ab,id.  6. ("internet of things" or "IoT").ti,ab,id.  7. Physical activity/ or Activity Level/ or Physical Fitness/  8. Blood Pressure/ or Heart Rate/ or Respiration/  9. (respirat* or "heart rate*").ti,ab,id.  10. Sleep/  11. ((sleep adj3 pattern) or (sleep adj3 cycle) or (sleep adj3 track)).ti,ab,id.  12. Technology/ or Navigation Technology/  13. exp Electroencephalography/  14. Aging/ or Gerontology/ or Geriatrics/  15. ("old* adult*" or elder* or (senior* not "high school") or aged or aging or ageing).ti,ab,id.  16. 14 or 15  17. Social isolation/ or Loneliness/  18. ("social isola*" or "social engag*" or "social connect*" or "social disconnect*" or "social support*" or lonel*).ti,ab,id.  19. 17 or 18  20. 1 or 2 or 3 or 4 or 5 or 6 or 7 or 8 or 9 or 10 or 11 or 12 or 13  21. 16 and 19 and 20  22. limit 21 to (human and english language) |
| IEEE  (((((("Document Title":"social isolation" OR "social engagement" OR "social connection" OR "social disconnection") OR "Abstract":"social isolation" OR "social engagement" OR "social connection" OR "social disconnection" OR lonely OR loneliness) OR "Author Keywords":"social isolation" OR "social engagement" OR "social connection" OR "social disconnection" OR lonely OR loneliness) OR "Publication Title":"social isolation" OR "social engagement" OR "social connection" OR "social disconnection" OR lonely OR loneliness))) |
